# Compliance With Neoadjuvant Chemotherapy in T4 Oral Cancers: Place, Person, Socioeconomic Status, or Assistance

**DOI:** 10.1200/JGO.2015.000265

**Published:** 2015-10-28

**Authors:** Vijay M. Patil, Vanita Noronha, Amit Joshi, Vamshi Muddu, Sachin Dhumal, Atanu Bhattacharjee, Kumar Prabhash

**Affiliations:** **Vijay M. Patil, Vanita Noronha, Amit Joshi, Vamshi Muddu, Sachin Dhumal, and Kumar Prabhash,** Tate Memorial Hospital, Mumbai; **Atanu Bhattacharjee,** Malabar Cancer Centre, Kerala, India

## Abstract

**Purpose:**

Approximately 15% to 20% of our patients with head and neck cancer receiving neoadjuvant chemotherapy (NACT) discontinue therapy because of various nonmedical reasons. We sought to analyze the factors associated with treatment default and noncompliance among these patients.

**Patients and Methods:**

We performed a retrospective analysis of patients with T4 oral cancer treated with NACT between January 2011 and December 2012. We included patients who discontinued treatment for nonmedical reasons before the second cycle of NACT. The factors analyzed were income, education, socioeconomic status, age, sex, place of residence, habits, and payment pattern (government supported or personal capacity). Pearson χ^2^ test was used to identify significant factors associated with noncompliance.

**Results:**

Of 486 patients, 91 patients (18.7%) were noncompliant. Percentages of noncompliant patients in the age groups < 30, between 30 and 60, and > 60 years were 25.0%, 17.4%, and 25.5%, respectively (*P* = .27). Percentages of noncompliance in patients residing within the city, same state, or different state were 20.7%, 20.9%, and 17.1%, respectively (*P* = .44). Noncompliance rates were 20.3%, 15.7%, 18.1%, and 22.5% in upper middle, lower middle, upper lower, and lower economic strata, respectively (*P* = .60). Similarly, there was no significant difference in noncompliance according to occupation, education level, payment pattern, sex, or habits.

**Conclusion:**

Our analysis failed to identify any specific significant factor associated with noncompliance with NACT among our patients with T4 oral cancers.

## INTRODUCTION

Neoadjuvant chemotherapy (NACT) is one modality of treatment in locally advanced head and neck cancers.^1–3^ Recently, we analyzed and published the results of NACT in patients with technically unresectable oral cancers.^4^ Although two cycles of chemotherapy were planned for all patients, we found that 15.8% of patients discontinued treatment after the first cycle of chemotherapy and did not receive the second cycle. These patients did not receive any further curative treatment and had a dismal prognosis.^4^ Recently published results of the PARADIGM study^5^ and a randomized trial of NACT by Hitt et al^6^ showed similar discontinuation rates. The noncompliance rate (discontinuation of NACT before local treatment) was 10% in PARADIGM and much higher (31.6%) in the study reported by Hitt et al. In India, the rate of noncompliance with curative treatment in head and neck cancers reported at a tertiary care center in New Delhi was as high as 38%.^7^ Noncompliance is a serious issue that can compromise treatment and lead to suboptimal outcomes.

In India, oral cancer is a common malignancy and is predominantly seen in men from lower socioeconomic classes, a majority of whom present to the hospital with locally advanced disease.^8,9^ The management of such patients requires a multimodal approach and is a resource intensive exercise.^1,2^ In our country, there are various social, economic, and logistic issues that may significantly affect the treatment of cancer.^10^ A majority of the population resides in the rural areas, whereas cancer centers are disproportionately located in cities; affordable health care thus entails significant travel.^10,11^ Added to rising health care costs, patients often find it difficult to complete long treatments. The high noncompliance rate in a potentially curative setting puts a strain on the available infrastructure. Identifying factors associated with noncompliance could help in better selection of patients, more rational use of limited resources, and improvement of treatment outcomes.

## PATIENTS AND METHODS

### Patient Selection

We maintain a database of all patients who have received NACT for oral cancer. Study patients were selected from this database, and the data were updated using electronic medical records. The study protocol was approved by the institutional review board.

#### Inclusion criteria.

Included patients had biopsy-proven T4 squamous cell cancer of the oral cavity, had technically unresectable tumors (ie, high probability of R1 or R2 resection), were unsuitable for radical concurrent chemoradiation (ie, unlikely to tolerate because of extensive locoregional disease), had Eastern Cooperative Oncology Group performance status of 0 to 1, or were intended to receive NACT under any protocol and had received ≥ one cycle of therapy.

#### Exclusion criteria.

Patients were excluded if they stopped NACT after the first cycle because of disease progression or intolerable adverse effects.

**Figure 1:**
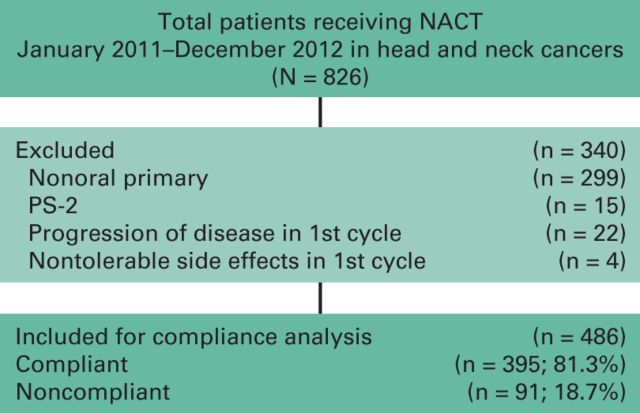
CONSORT diagram: patient selection criteria. NACT, neoadjuvant chemotherapy; PS-2, Eastern Cooperative Oncology Group performance status-2.

Figure 1 provides a detailed description of patient selection. Data are shown for patients treated between January 2011 and December 2012. All patients were first seen in a multidisciplinary joint clinic and were intended to receive two cycles of NACT followed by reassessment for surgery. Depending on response and patient performance status, intent of treatment (curative *v* palliative) and further therapy were decided at a multidisciplinary clinic. The NACT protocol was selected considering a patient's medical comorbidities, performance status, and logistic issues, including financial capacity and place of residence. We have discussed the selection criteria, chemotherapy protocols, and further treatment offered in detail in a previous publication.^4^

#### Noncompliance.

For this analysis, any patient who received one cycle of NACT but then discontinued further treatment was considered noncompliant, except for those patients for whom NACT was stopped because of medical reasons, as outlined in the exclusion criteria.

### Selection of Factors Affecting Compliance

Factors affecting compliance were selected on the basis of literature review and consensus decision after discussion between authors (V.M.P., V.N., A.J., A.B., and K.P.).^10,12–16^ Factors considered potentially significant for determining noncompliance and the rationale behind selecting these factors are detailed as follows:

#### Patients age at diagnosis.

Age was tested as a categorical variable. We considered three age groups: ≤ 30, between 30 and 60, and > 60 years.

#### Sex.

This factor was selected because sociocultural conditions might lead to bias against women in completing treatment.

#### Education.

The education level of the patient was scored in accordance with modified Kuppuswamy scale.^17^ It was tested as a categorical variable.

#### Patients income.

The monthly family income was scored in accordance with modified Kuppuswamy scale.^17^

#### Place of residence.

A patient's place of residence was classified as local (patient staying in main town, suburb, or area covered by local transport facilities), regional (patient staying within state but not qualifying for definition of local), or distant (patient staying outside state).

#### Local social support.

In India, family support is often essential for successfully completing therapy. Patients with availability of relatives or friends in the locality were considered as having local support. This factor was tested as a categorical variable.

#### Addiction.

Patients with addictions may find it difficult to continue their habits under constant supervision of the medical team and family members. This variable was tested as categorical variable.

#### No government support for treatment.

Patients not receiving financial assistance for treatment from the government may find it difficult to support their therapy and hence may stop. This factor was tested as a categorical variable.

#### Socioeconomic status in accordance with modified Kuppuswamy status.

Socioeconomic stratification was performed according to the modified Kuppuswamy scale, a socioeconomic scale first published in 1979 for urban populations.^17^ An individual score is first assigned for family income, occupation, and education, after which a cumulative score is generated. Although the scores for education and occupation remain the same, the score for income is dynamic. We used the modification published by Bairwa et al.^17^ The income score was calculated after adjusting for inflation until 2012. This classifies patients into five strata: upper, upper middle, lower middle, upper lower, and lower.

### Statistical Analysis

Analysis was performed using RStudio software (version 3.1.2; R Project, Vienna, Austria). Descriptive statistics were performed. The categorical factors were tested using Pearson's χ^2^ test with Yates' continuity correction. The dependent variable was noncompliance.

## RESULTS

### Baseline Details

Over the stipulated period, 826 patients received NACT for head and neck cancer. A total of 486 patients were eligible for this study. The CONSORT diagram shows reasons for patient exclusion and details of treatment (Fig 1). Overall, in the total cohort selected for this analysis, 91 patients (18.7%) were noncompliant with NACT.

The median age of patients was 49 years (interquartile range, 39 to 55 years). The numbers of men and women were 405 (83.3%) and 81 (16.7%), respectively. The level of education, income, occupation, social support details, government financial assistance details, place of residence, and details of addictions are listed in Table 1. A majority of patients (n = 268 [55.1%]) had education levels below middle school (ie, seventh grade). The median income was 2,000 INR (US$30.76). According to the modified Kuppuswamy scale, 102 patients (21.0%) were in the lower socioeconomic group. A majority of patients (n = 404 [83.1%]) were from a different city in the same or a different state. Local social support and government assistance schemes were available for only 183 (37.7%) and four patients (0.8%), respectively.

**Table 1:** Distribution of Factors Considered for Noncompliance With NACT (N = 486)

Factor	No. of Patients (%)
Age group, years	
< 30	32 (6.6)
30 to 60	402 (82.7)
> 60	51 (10.7)
Education level	
Professional or honors	0 (0.0)
Graduate or postgraduate	90 (18.5)
Intermediate or post–high school diploma	0 (0.0)
High school certificate	128 (26.3)
Middle school certificate	56 (11.5)
Primary school certificate	119 (24.5)
Illiterate	93 (19.2)
Occupation	
Professional	8 (1.6)
Semiprofessional	84 (17.3)
Clerk, shop owner, or farmer	130 (28.9)
Skilled worker	7 (1.4)
Semiskilled worker	16 (3.3)
Unskilled worker	55 (11.3)
Unemployed	176 (36.2)
Income	
≥ 31,507 INR (US$485.72)	0 (0.0)
15,754 to 31,506 INR (US$242.36 to US$484.71)	1 (0.2)
11,817 to 15,753 INR (US$181.79 to US$242.35)	64 (13.2)
7,878 to 11,816 INR (US$121.19 to US$181.78)	48 (09.9)
4,727 to 7,877 INR (US$72.72 to US$121.18)	93 (19.2)
1,590 to 4,726 INR (US$24.46 to US$72.71)	121 (24.9)
≤ 1,589 INR (≤ US$24.45)	159 (32.6)
Socioeconomic stratum*	
Upper	0 (0.0)
Upper middle	64 (13.2)
Lower middle	115 (23.7)
Upper lower	205 (42.1)
Lower	102 (21.0)
Local social support	
Present	183 (37.7)
Absent	303 (62.3)
Government assistance schemes	
Present	4 (0.8)
Absent	482 (99.2)
Place of residence	
City in which hospital is located (local)	82 (16.9)
State in which hospital is located (regional)	129 (26.5)
Outside state but within country (distant)	275 (56.6)
Habits	
History of tobacco, illicit drug, or alcohol use	464 (90.5)
No history of tobacco, illicit drug, or alcohol use	22 (9.5)

Abbreviation: INR, Indian rupee; NACT, neoadjuvant chemotherapy.

* According to modified Kuppuswamy scale.

Regarding clinical characteristics, tumor stage was T4a in 405 patients and T4b in 81 patients. Nodal stage was N2 in 444 patients and N3 in 42 patients. Type of NACT used was a three-drug regimen (docetaxel, cisplatin, and fluorouracil) in 30 patients (6.7%) and a combination of two drugs (taxane plus platinum) in 456 patients (93.3%).

### Factors Associated With Compliance

#### Place of residence.

A majority of patients were from outside the state in which our center is located. However, place of residence was not associated with noncompliance. As summarized in Table 2, noncompliance percentages were similar irrespective of whether patients resided in the same city, the same state, or a distant place (20.7%, 20.9%, and 17.1%, respectively; *P* = .44).

**Table 2:** Relationship Between Place of Residence and Noncompliance

Place of Residence	No. of Patients	No. of Noncompliant Patients (%)
Local (city in which hospital is located)	82	17 (20.7)
Regional (state in which hospital is located)	129	27 (20.9)
Distant (outside state but within country)	275	47 (17.1)

NOTE. Pearson's χ^2^ test with Yates' continuity correction was used for comparison (χ^2^, 0.6026; *df*, 1; *P* = .44).

#### Patient demographics.

A majority of patients were in the age group between 30 and 60 years (n = 402 [82.7%]). Only 22 patients were without any previous history of addiction, as defined by tobacco, alcohol, or intravenous drug abuse. As listed in Table 3, there was no statistical difference in noncompliance rate in the different subdivisions of age or habits. Percentages of patients with noncompliance in age groups < 30, between 30 and 60, and > 60 years were 25.0%, 17.4%, and 25.5%, respectively (*P* = .27).

**Table 3:** Relationship Between Personal Factors and Noncompliance

Variable	No. of Patients	No. of Noncompliant Patients (%)	*P*
Age group, years			
< 30	32	8 (25.0)	.40 (< 30 *v* 30 to 60 years)
30 to 60	402	70 (17.4)	.40 (< 30 *v* > 60 years)
> 60	51	13 (25.5)	1 (> 60 *v* 30 to 60 years)
Habits			
History of tobacco, illicit drug, or alcohol use	464	85 (18.3)	.44
No history of tobacco, illicit drug, or alcohol use	22	6 (27.3)	χ^2^, 0.5964; *df*, 1

NOTE. Pearson's χ^2^ test with Yates' continuity correction was used for comparison.

#### Dependence of compliance on socioeconomic status.

Noncompliance distributions according to modified Kuppuswamy socioeconomic scale were 20.3%, 15.7%, 18.1%, and 22.5% in upper middle, lower middle, upper lower, and lower strata, respectively. There was no statistically significant difference in this distribution. Similarly, there was no difference in noncompliance rate according to the modified Kuppuswamy socioeconomic strata of occupation, education, or income (Data Supplement).

#### Dependence of compliance on assistance.

Availability of social support and financial assistance schemes did not affect the compliance rate. Percentages of noncompliant patients with and without local family support were 20.8% (38 of 183 patients) and 17.5% (53 of 303 patients), respectively (*P* = .43). Although there was 0% noncompliance among patients receiving government schemes, the number of patients (n = 4) was too low to reach any conclusions.

## DISCUSSION

Compliance with cancer treatment is a major issue in India and other countries in the developing world.^4,7,14,18–20^ As an illustrative example, reported incidences of nonadherence to curative treatment in head and neck cancers and lung cancer were 38% and 68.3%, respectively, in a premier hospital in India.^7,21^ The reported incidence of noncompliance with management of adult hematologic malignancies has also varied from 16% to 71%.^22,23^ Similarly, the no compliance rate for pediatric solid tumor treatment in India is alarmingly high, ranging from 10% to 62% in different cancer centers across the country.^24^ Noncompliance rates in other developing countries in Asia, central America, and Africa for pediatric solid tumor treatment have ranged from 4% to 67%, 1% to 41%, and 4% to 50%, respectively.^24^ Poor compliance and adherence to treatment are associated with poor survival rate.^23,25–27^ In our analysis, the noncompliance rate for NACT was 18.7%.

Multiple factors that potentially affect compliance have been discussed in various studies and were tested in our study.^19,20,28–33^ Age is an important consideration in various settings. Patients of younger ages, especially adolescent males, have been shown to be at risk for noncompliance with treatment.^18,20^ Similarly, patients of younger ages have increased nonadherence to breast cancer treatment in Africa.^26^ Patients with addictions (especially to alcohol) are known to be noncompliant with medical management, commonly in tuberculosis treatment.^29,32^ Financial issues often dictate compliance in developing countries, as shown in a recent study from Malaysia, in which only 19% of patients could afford trastuzumab.^34^ The corresponding figure from India is a dismal 4.1%.^35^ Socioeconomic stratum, level of education, prolonged travel time to treatment facility, and younger age are other known factors associated with nonadherence to acute leukemia treatment in low- and middle-income countries.^22–24,27^ Nonavailability of adequate social or family support and nonavailability of government or public assistance schemes are additional factors associated with noncompliance with treatment. We wanted to test all these factors in our patient population receiving NACT for head and neck cancers. Considering our patient load and limited resources, we cannot formulate a uniform support system for all patients. The goal was to identify a specific subgroup likely to have a higher chance of nonadherence to therapy. We could then formulate targeted approaches and offer resources like social workers, financial incentives, and residential amenities to ensure completion of therapy.

Rather surprisingly, our analysis failed to identify any factor that could be significantly associated with noncompliance. This was despite the illiteracy and unemployment rates being higher in our patients than in the general population according to census data (illiteracy rate, 19.2% *v* 11.3%; unemployment rate, 36.2% *v* 7.6%).^36,37^ One potential reason could be that patients agreed to therapy after a detailed discussion regarding the logistics and financial issues with the medical oncologist. This counseling session could have acted as a social selection process that nullified the effects of some of the tested factors. Second, there might be unaddressed factors like underlying distress, psychological issues, communication gap, lack of bonding, or lack of motivation that could contribute to noncompliance. Patient distress especially has been related to a higher rate of noncompliance with medical advice.^38^ A study by Dessai et al^39^ at a rural Indian cancer center highlighted the high rates of actionable distress (41.0%) among patients with cancer. The patient-to-physician ratio is poor in India, affecting the amount of time spent on communication.^11^ Lack of adequate communication could lead to a lack of confidence and bonding with the treating physician; these factors have been shown to be associated with high rates of noncompliance in patients without cancer.^16,40^

There are limitations in our analysis. The primary problem seems to be the retrospective nature of the data. We could not perform any telephonic interviews or arrange for follow-up visits to ascertain the true causes of noncompliance. The other shortcoming may be an inability to correctly capture data regarding employer assistance schemes and financial status. Although a majority of patients have to finance their own treatment, several patients belong to a joint family system and have access to combined family income and inheritances that may not be adequately represented by a single factor like the income of the family head. These factors may have negated the impact of reported income and socioeconomic stratification.

Although the study is essentially negative, there is still an important message that commonly considered demographic and socioeconomic factors may not be the only contributing reasons behind noncompliance. There is a more complex interplay of causes, and efforts targeted against traditional shortcomings might not be enough to improve compliance.

To improve our compliance rate, we have decided to adopt a system similar to that proposed by Rosenberg et al.^41^ It is a multistep method that will help us identify patients at high risk for noncompliance and takes into account not only the socioeconomic and demographic factors but also the psychosocial, physical, and intellectual factors. Every patient is now informed about the availability of assistance schemes, and a checklist is kept to ensure that all such options have been explained. A dedicated medical social worker is now available for patients with head and neck cancer to assist with any logistic issues, including finances and residence. A support group for patients with head and neck cancer is being established with the aim of counseling and providing motivation for new patients. In addition, our clinical case sheet has been modified to capture data regarding socioeconomic stratification. We plan to study the compliance rate after 1 year to see if there is any improvement after these measures.

In conclusion, our analysis failed to identify any specific factor associated with noncompliance among patients with head and neck cancer receiving NACT. Established factors like socioeconomic stratum, education level, income, profession, age, sex, social support, and financial assistance schemes did not have a significant impact in our patient population. Our current endeavor is to increase the support staff and put in place a more structured program to improve the overall compliance rate.
